# Nitrogen Removal of the Sunlight-Irradiated SBABR and Anammox Combined System: Performance and Mechanism

**DOI:** 10.3390/microorganisms14020435

**Published:** 2026-02-12

**Authors:** Zichun Yan, Yuyang Han, Zhibin Pei, Shuichao Fan

**Affiliations:** 1 School of Environmental and Municipal Engineering, Lanzhou Jiaotong University, Lanzhou 730070, China; hanyuyang0326@outlook.com (Y.H.); peizhibin2025@outlook.com (Z.P.); fanshuichao317@outlook.com (S.F.); 2 Key Laboratory of Yellow River Water Environment of Gansu Province, Lanzhou 730070, China

**Keywords:** partial nitrification, anaerobic ammonium oxidation, sunlight exposure inhibits NOB activity, chlorophyll-a removal, microbial community structure

## Abstract

To develop a low-energy nitrogen removal system, a coupled system consisting of a sunlight-enhanced bacteria-algae biological rotor (SBABR) and an anaerobic ammonia oxidation (Anammox) reactor was constructed. This study investigated partial nitrification stability in the SBABR reactor, color and chlorophyll-a removal efficiency in the Anammox reactor, and the coupled system’s nitrogen and carbon removal performance and microbial community structure using high-throughput sequencing. Under significant light intensity fluctuations (300–2400 μmol/(m^2^·s)), the SBABR reactor maintained nitrite accumulation rates (NAR) of 94.98–98.13% with effluent NO_2_^−^-N/NH_4_^+^-N ratios between 1.01 and 1.18. The coupled system achieved average total nitrogen (TN) removal efficiency of 81.78% and ammonia nitrogen (NH_4_^+^-N) removal efficiency of 87.71%. SBABR effluent color decreased from 42 to 17 Hazen units after the Anammox reactor, and chlorophyll-a concentration dropped from 296.40 μg/L to below detection limits. Microbial community analysis revealed that *Nitrosomonas* abundance was 0.69%, while *Nitrospira* abundance was only 0.01% in the SBABR reactor. In the Anammox reactor, Planctomycetota abundance reached 13.30%, and *Candidatus Jettenia* reached 11.57%. Results indicate that the activity of both AOB and NOB is inhibited by sunlight, but the inhibition of NOB is more significant, enabling stable nitrite accumulation. The Anammox reactor demonstrated favorable nitrogen removal performance and effectively removed color and chlorophyll-a.

## 1. Introduction

With the acceleration of global industrialization and urbanization, substantial nitrogen-containing pollutants are discharged into water bodies, leading to eutrophication [1,2]. Currently, nitrification–denitrification processes are primarily employed to treat nitrogen pollutants in wastewater; however, these processes require considerable oxygen and carbon sources, resulting in high energy consumption and operational costs [3]. Therefore, developing low-energy nitrogen removal processes holds significant environmental and economic importance.

The partial nitrification–anaerobic ammonium oxidation (PN/A) process, as an emerging biological nitrogen removal technology [4], first oxidizes a portion of ammonium (NH_4_^+^) to nitrite (NO_2_^−^) through partial nitrification, then utilizes AnAOB to directly convert NH_4_^+^ and NO_2_^−^ to nitrogen gas (N_2_) [5,6]. Compared to conventional processes, the PN/A process reduces oxygen demand by approximately 60%, without requiring external carbon sources, and decreases sludge production by approximately 80% [7]. However, achieving stable partial nitrification is the primary challenge for the PN/A process [8], typically requiring precise control of operating conditions to inhibit NOB activity. For instance, partial nitrification has been achieved through heating and intermittent aeration [9,10]; utilizing the higher activity of AOB compared to NOB under low dissolved oxygen (DO) conditions to suppress NOB [11] and exploiting the inhibitory effects of free ammonia (FA) and free nitrous acid (FNA) on nitrite-oxidizing bacteria to achieve partial nitrification [12].

The aforementioned methods for achieving partial nitrification present inherent limitations. Research has indicated that heating often requires more energy consumption, while intermittent aeration necessitates complex operational systems [13], thereby increasing operational costs. Although low dissolved oxygen conditions can achieve partial nitrification in activated sludge systems, NOB can adapt to low DO conditions during long-term operation, leading to destabilization of partial nitrification [14]. Wang et al. discovered that the FA inhibition method for achieving partial nitrification failed when the influent NH_4_^+^-N concentration was reduced to 100 mg/L [15]. Consequently, finding more effective, operationally simple, and energy-efficient methods for achieving partial nitrification is crucial.

Research has confirmed that light exerts stronger inhibition on NOB than on AOB, effectively promoting partial nitrification [16]. Si et al. utilized blue-light illumination to increase the nitrite accumulation rate to over 90% within 15 days [17]. Our research group employed LED illumination in rotating biological contactors to promote bacteria–algae consortium formation, achieving over 90% nitrite accumulation rate in low C/N wastewater treatment [18]. Most current studies employ artificial light sources, with relatively limited research on utilizing sunlight to establish partial nitrification [19,20,21,22].

Following light exposure, activated sludge systems often transform into bacteria-algae symbiotic systems as algae proliferate [23]. As bacteria–algae biofilms slough off, increased effluent turbidity and color can cause secondary pollution [24], posing significant challenges for practical system applications. However, color issues in bacteria–algae symbiotic partial nitrification–Anammox coupled systems have not yet been investigated [25,26,27,28].

Based on these considerations, this study employed sunlight as the light source to achieve partial nitrification on slowly rotating biological contactors, connected the biological contactor effluent to an Anammox reactor to accomplish low-energy nitrogen removal, and simultaneously removed color and chlorophyll-a from the SBABR reactor effluent.

## 2. Materials and Methods

### 2.1. Synthetic Wastewater and Seed Sludge

The synthetic wastewater composition was as follows: NH_4_^+^-N 240 ± 10 mg/L, COD 205 ± 5 mg/L, and sodium bicarbonate (NaHCO_3,_ Baishi Chemical Co., Ltd., Tianjin, China) 1500 mg/L, with trace elements including calcium chloride (CaCl_2,_ Baishi Chemical Co., Ltd., Tianjin, China) 20 mg/L, potassium dihydrogen phosphate (KH_2_PO_4,_ Baishi Chemical Co., Ltd., Tianjin, China) 20 mg/L, and magnesium sulfate heptahydrate (MgSO_4_·7H_2_O, Baishi Chemical Co., Ltd., Tianjin, China) 20 mg/L. The rotating biological contactor was seeded with aerobic activated sludge from a wastewater treatment plant in Lanzhou, with a seeding volume of 10 L. The Anammox reactor was seeded with stable anaerobic ammonium oxidation sludge, with a seeding volume of 6.5 L.

### 2.2. Experimental Setup

**Figure 1:**
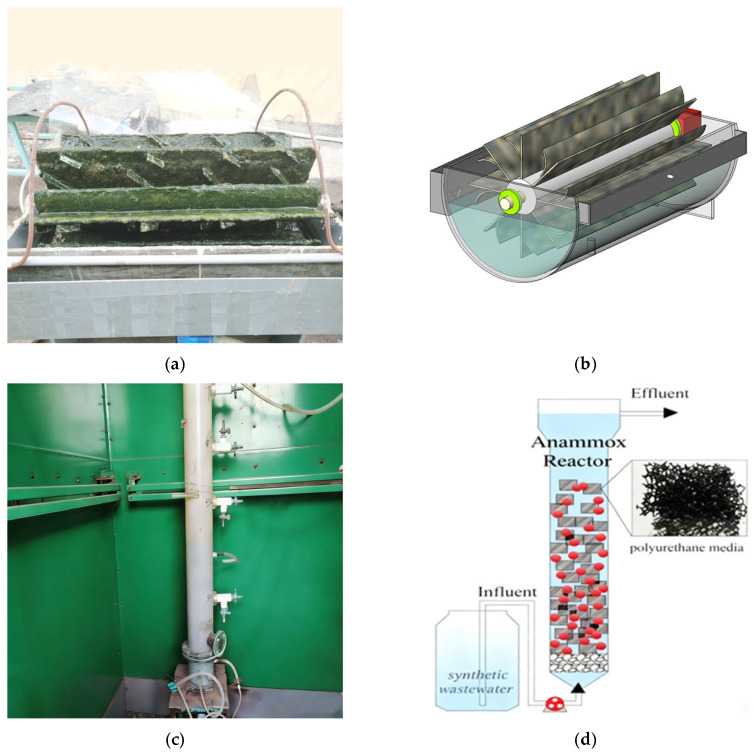
Experimental apparatus: (**a**) Photograph of SBABR reactor; (**b**) Schematic diagram of SBABR reactor; (**c**) Photograph of Anammox reactor; (**d**) Schematic diagram of Anammox reactor.

As shown in Figure 1a, the main body of the SBABR reactor had a diameter of 40 cm and a length of 65 cm, with an effective volume of 28 L, comprising a semi-circular contact reaction tank, rotating disks, influent/effluent weirs, and a transmission device. As illustrated in Figure 1b, the rotating disk assembly comprises eight sets of spokes, each featuring a straight plate and a curved plate configured in a ‘y’ shape to enhance microbial light exposure through optimized spatial orientation and increased effective surface area for sunlight capture. The surface of each plate is covered with a 2 mm thick polyester sponge. The reactor employed a horizontal flow pattern, with the disk rotation direction opposite to the influent flow direction. The reactor and feed water tank were placed in an insulated chamber to maintain stable water temperature. Influent of the Anammox reactor was the effluent from the SBABR reactor(The SBABR was fabricated in-house at the School of Environmental and Municipal Engineering, Lanzhou Jiaotong University, Lanzhou, China.). The water was heated during storage in the feed tank and during conveyance into the Anammox reactor, reaching 30 ± 2 °C upon entry. As shown in Figure 1c, the Anammox biofilm reactor utilized an upflow acrylic column with an effective volume of 14.9 L, an inner diameter of 10 cm, a carrier-bed height of 0.86 m, and a total column height of 2.1 m. As illustrated in Figure 1d, the column was packed with polyurethane media measuring 1.5 × 1.5 × 2.5 cm.

### 2.3. Experimental and Analytical Methods

#### 2.3.1. Water Quality Analysis and Determination Methods

TN concentration was measured using a UV spectrophotometer (DR5000, HACH, Loveland, CO, USA). NH_4_^+^-N, NO_2_^−^-N, and NO_3_^−^-N concentrations were measured using a UV–visible spectrophotometer (721G Visible Spectrophotometer, INESA, Shanghai, China). TN, NH_4_^+^-N, NO_2_^−^-N, and NO_3_^−^-N were measured according to standard methods [29]. COD concentration was measured using a UV–visible spectrophotometer (DR5000, HACH, Loveland, CO, USA) by the potassium dichromate colorimetric method [30]. pH, temperature (T), and DO were measured directly using a water-quality analyzer (HQ-10, HACH, Loveland, CO USA), with pH and DO requiring their respective pH probe and DO probe. Effluent color was determined using the platinum–cobalt colorimetric method [31]. Chlorophyll-a was measured using the acetone extraction method [32].

#### 2.3.2. FA and FNA Calculation Methods

*FA* and *FNA* concentrations were calculated according to the following equations [33]:

(1) $$FA=\frac{17}{14}\frac{C_{\left[{{\mathrm{NH}}_{4}}^{+}-N\right]}\times 10^{pH}}{\exp\left(\frac{6334}{273+T}\right)+10^{pH}},$$

(2) $$FNA=\frac{46}{14}\frac{C_{\left[{{\mathrm{NO}}_{2}}^{-}-N\right]}}{\exp\left(-\frac{2330}{273+T}\right)+10^{pH}}$$

where $C_{\left[NH_{4}^{+}-N\right]}$ is the concentration of NH_4_^+^-N, $C_{\left[NO_{2}^{-}-N\right]}$ is the concentration of NO_2_^−^-N, and *T* is temperature (°C).

#### 2.3.3. Microbial Analysis Methods

This study employed 16S rRNA high-throughput sequencing technology to investigate the bacterial community structure in the system [34]. Bacterial samples from the SBABR were designated as PN, while samples from the Anammox reactor were designated as AN. Samples were sent to Majorbio Bio-pharm Technology Co., Ltd. (Shanghai, China) for analysis.

Total microbial genomic DNA was extracted from samples using the E.Z.N.A.^®^ Soil DNA Kit (Omega Bio-tek, Norcross, GA, USA) according to the manufacturer’s instructions. The hypervariable regions V3-V4 of the bacterial 16S rRNA gene were amplified using the primer pair 338F (5′-ACTCCTACGGGAGGCAGCAG-3′) and 806R (5′-GGACTACHVGGGTWTCTAAT-3′). Paired-end sequencing was performed on the Illumina PE300/PE250 platform (Illumina, San Diego, CA, USA) according to the standard protocols of Majorbio Bio-pharm Technology Co., Ltd. (Shanghai, China).

## 3. Results

### 3.1. Bacteria–Algae Symbiotic Partial Nitrification Performance

During the experimental period, the SBABR reactor utilized sunlight as the light source with an average light intensity of 1275 μmol/(m^2^·s). Light duration varied seasonally, and the hydraulic loading rate was 0.35 m^3^/(m^2^·d) and influent pH of 8 ± 0.5. To analyze the microbial-community structure, bacterial samples from the SBABR reactor were designated as PN and subjected to high-throughput sequencing analysis.

**Figure 2:**
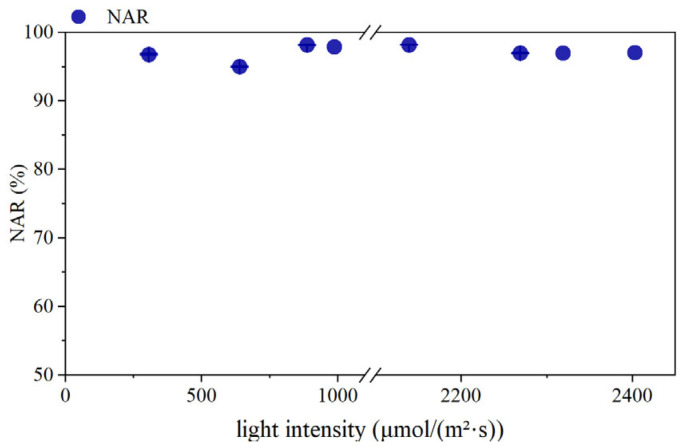
NAR variation with light intensity.

During the experimental period, light intensity varied with weather conditions. To investigate the effects of different light conditions on partial-nitrification performance, corresponding light intensity and NAR values were monitored. As shown in Figure 2, the monitoring period included two rainy days (light intensity below 640 μmol/(m^2^·s)), while the remaining days were sunny days (light intensity above 2000 μmol/(m^2^·s)) and cloudy days (light intensity between 700 and 1000 μmol/(m^2^·s)). As shown in Figure 2, the light-intensity range for the selected experimental days was 300–2400 μmol/(m^2^·s). Despite such substantial light intensity variations, the system NAR remained consistently high, maintaining above 95%, demonstrating that the SBABR reactor achieved stable and efficient partial nitrification.

**Figure 3:**
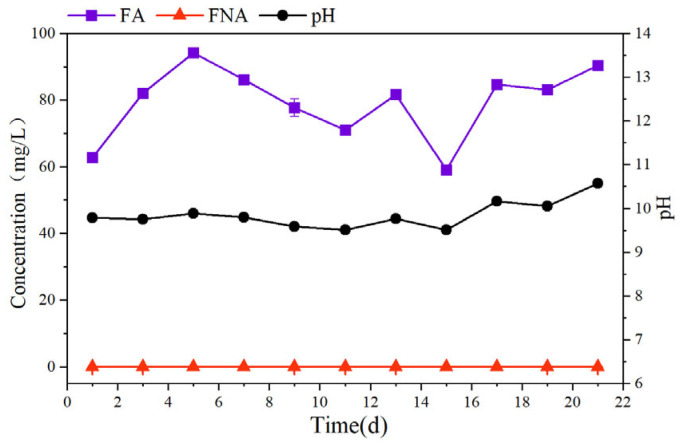
FA and FNA Concentrations and pH in the SBABR Reactor.

As illustrated in Figure 3, algal photosynthesis consumed CO_2_ in the water, increasing the system pH to an average value of 9.86, resulting in high FA concentrations with an average of 80.87 mg/L. The inhibition threshold of FA for NOB (0.1–1 mg/L) is significantly lower than that for AOB (10–150 mg/L) [35], resulting in more substantial inhibitory effects of high FA on NOB. Additionally, algae enhanced dissolved-oxygen availability in the microenvironment through photosynthesis, providing supplementary oxygen to support the nitrification process [36].

**Figure 4:**
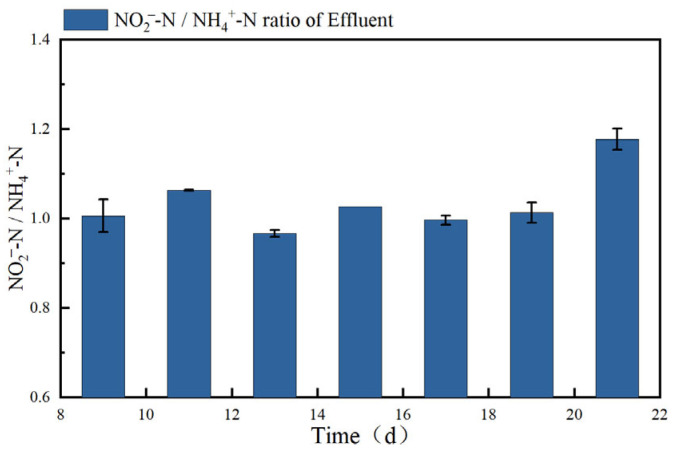
Effluent NO_2_^−^-N/NH_4_^+^-N ratio during stable partial nitrification.

**Figure 5:**
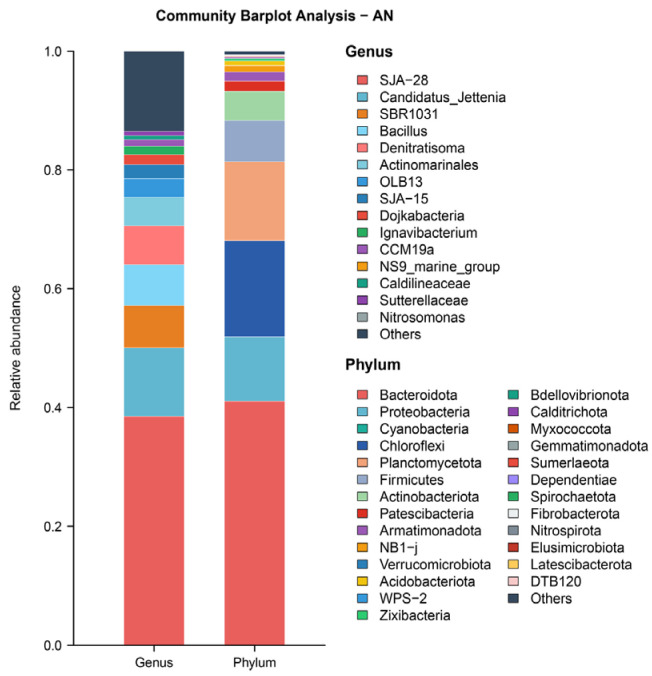
Microbial-community abundance in Anammox reactor.

As shown in Figure 4, the average effluent NO_2_^−^-N/NH_4_^+^-N ratio from the SBABR reactor during stable operation was 1.04. As indicated in Figure 5, the abundance of *Bacillus* accounted for 6.89%. Some microorganisms within the *Bacillus* genus possess dissimilatory nitrate reduction capabilities, enabling them to reduce NO_3_^−^ to NO_2_^−^ under anoxic conditions [37], providing additional electron acceptors for the anammox process. This explains why, although the SBABR reactor effluent NO_2_^−^-N/NH_4_^+^-N ratio was lower than the theoretical value, favorable anammox performance was still achieved, consistent with findings reported by Yang et al. [38].

**Figure 6:**
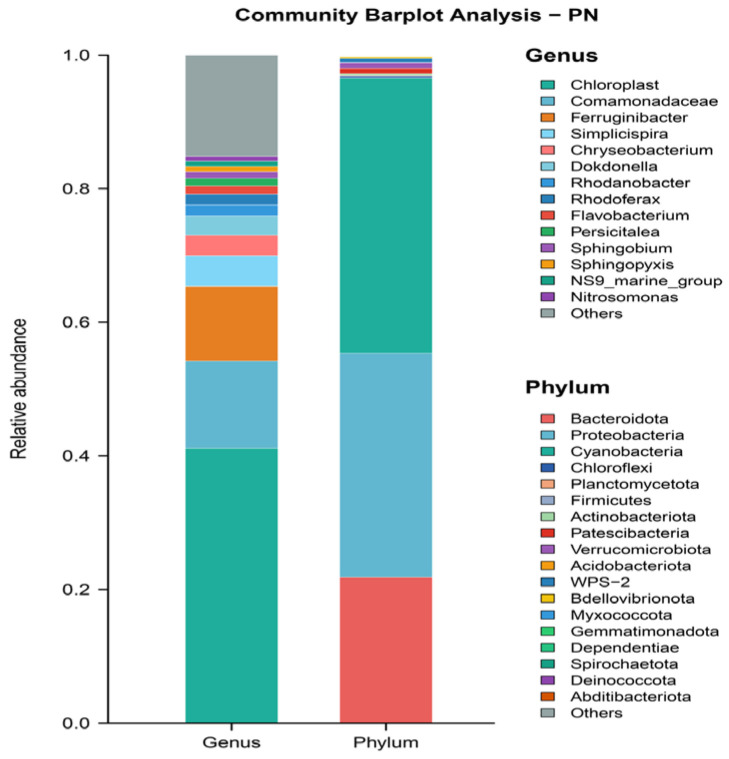
Microbial-community abundance in SBABR reactor.

As shown in Figure 6, microbial community analysis revealed distinct compositional characteristics in the SBABR reactor. At the phylum level, the relative abundance of Cyanobacteria reached 41.22%, while at the genus level, chloroplast sequences accounted for 41.11% of the relative abundance. These data indicated that under sunlight irradiation, a photosynthetic algae-dominated bacteria–algae symbiotic system was established within the reactor. Algae consumed carbon dioxide in the water through photosynthesis [39], resulting in an increase in system pH to an average of 9.86 and free ammonia concentration of 80.87 mg/L, thereby providing favorable conditions for achieving stable partial nitrification. As shown in Figure 6, analysis at the genus level revealed that in the SBABR reactor samples, *Nitrosomonas*, representing AOB [40], accounted for 0.69% abundance, while *Nitrospira*, the primary representative of NOB [41], accounted for only 0.01% abundance.

As shown in Table 1, the *Nitrosomonas* to *Nitrospira* abundance ratio reached 69, which was higher than the ratios observed in rotating biological contactors without sunlight. This high *Nitrosomonas* to *Nitrospira* abundance ratio (69:1) provided the biological foundation for excellent partial nitrification performance, ensuring NO_2_^−^ accumulation in the SBABR reactor and providing suitable substrates for subsequent anammox processes. Additionally, this study found that both *Nitrosomonas* and *Nitrospira* abundances in the SBABR reactor decreased to varying degrees compared to rotating biological contactors without sunlight, indicating that light has inhibitory effects on nitrifying bacteria, consistent with findings reported by Vergara et al. [42].

**Table 1:** Comparison of *Nitrosomonas* and *Nitrospira* abundance in SBABR reactor.

Light Conditions	*Nitrosomonas* Abundance	*Nitrospira* Abundance	*Nitrosomonas* to *Nitrospira* Abundance Ratio	Reference
Without light	0.80%	1.10%	0.73	[43]
Without light	0.85%	1.00%	0.85	[44]
Without light	8.78% ^1^(1.95%) ^2^	10.46% ^1^(2.71%) ^2^	0.84 ^1^(0.72) ^2^	[45]
With light	0.69%	0.01%	69	This study

^1^ indicates warm-season data, ^2^ indicates cold-season data.

### 3.2. Pollutant-Removal Performance of the Coupled System

The SBABR reactor, originally operated indoors under LED illumination, was relocated outdoors to receive sunlight. The SBABR reactor operated at a hydraulic loading rate of 0.35 m^3^/(m^2^·d), with a disk rotation speed of 2 r/min and influent pH of 8 ± 0.5 and an average water temperature of 24.93 °C. The Anammox reactor temperature was maintained at 30 ± 2 °C, with a hydraulic loading rate of 5.61 m^3^/(m^2^·d), and influent pH varying with the SBABR reactor effluent. To analyze the microbial community structure, bacterial samples from the Anammox reactor were designated as AN and subjected to high-throughput sequencing analysis.

#### 3.2.1. Nitrogen Removal Performance

**Figure 7:**
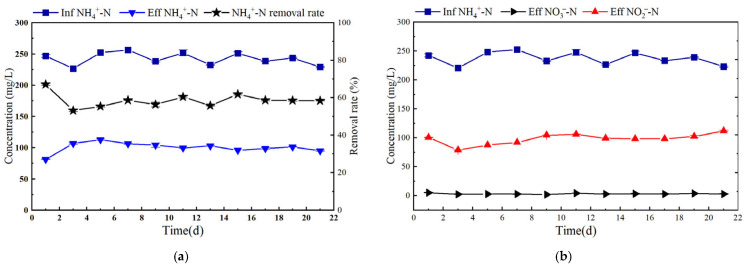
Nitrogen-removal analysis in SBABR reactor: (**a**) NH_4_^+^-N removal analysis; (**b**) Variations of Nitrogen Concentrations in Influent and Effluent.

As shown in Figure 7a, the ammonia nitrogen removal efficiency (ARE) of the SBABR reactor decreased from 67.06% to 53.05% within the first three days under sunlight, attributed to the sudden change in light source. After day 7, microorganisms adapted to sunlight and ARE gradually stabilized with an average value of 58.54%. As shown in Figure 7b, during the stable period, the average effluent NH_4_^+^-N concentration was 99.63 mg/L, and the NO_2_^−^-N concentration remained stable at an average of 103.02 mg/L.

**Figure 8:**
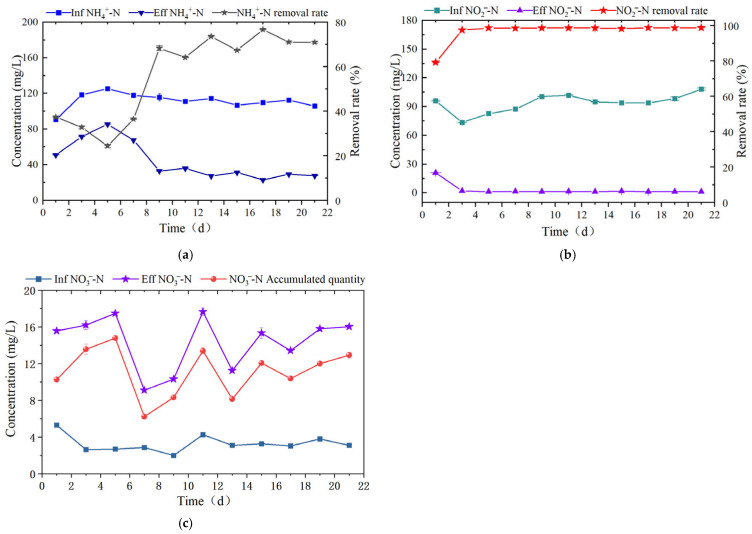
Nitrogen-removal analysis in Anammox reactor: (**a**) Variations in influent and effluent NH_4_^+^-N concentrations and removal rate; (**b**) Variations in influent and effluent NO_2_^−^-N concentrations and removal rate; (**c**) Variations in influent and effluent NO_3_^−^-N concentrations and accumulated quantity.

As illustrated in Figure 8a, the NH_4_^+^-N removal rate exhibited an initial decline followed by an increase, with an average removal rate of 70.27% during the stable operation period. The trend of initial decline followed by increase in NH_4_^+^-N removal rate was caused by the decreased ARE of the SBABR reactor. As shown in Figure 8b, the reactor achieved an average NO_2_^−^-N removal rate of 98.56%. As shown in Figure 8c, nitrate nitrogen (NO_3_^−^-N) was continuously generated as a byproduct of the Anammox process, with an average accumulation of 11.03 mg/L. The effluent concentrations of NH_4_^+^-N, NO_2_^−^-N, and NO_3_^−^-N were 29.62 mg/L, 1.32 mg/L, and 14.26 mg/L, respectively. The effluent NH_4_^+^-N concentration met the Integrated Wastewater Discharge Standard (GB 8978-1996) (this standard does not set limits for NO_2_^−^-N and NO_3_^−^-N) [46].

As shown in Figure 5, the relative abundances of Planctomycetota and *Candidatus Jettenia* were 13.30% and 11.57%. Planctomycetota represents a primary phylum of anammox bacteria [47], and *Candidatus Jettenia* represents a major genus of anammox bacteria [48]. These abundance levels indicate a successful enrichment of anammox bacterial communities in the reactor, which is consistent with the observed nitrogen-removal performance.

According to the chemical equation of the anammox reaction, when 1 mol NH_4_^+^ and 1.32 mol NO_2_^−^ are removed, 0.26 mol NO_3_^−^ is produced [49]. However, in this study, the ratio of NO_3_^−^ accumulation to NH_4_^+^ removal in the Anammox reactor was only 0.11, significantly lower than the theoretical value of 0.26. This was attributed to denitrification processes that consumed partial nitrate. As shown in Figure 8, analysis at the phylum level revealed that Proteobacteria accounted for 10.91% of the total abundance, primarily comprising denitrifying bacteria [50]. At the genus level, *Denitratisoma* accounted for 2.85% abundance, possessing denitrification capabilities [51].

**Figure 9:**
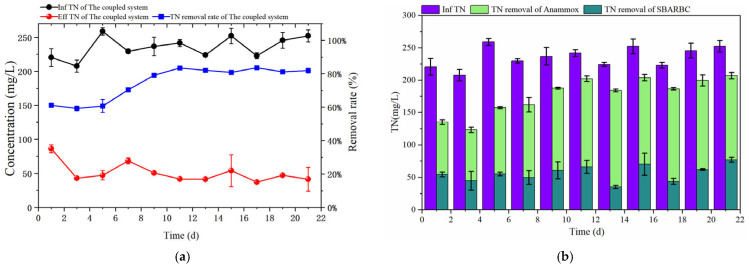
TN-removal analysis in the coupled system: (**a**) Variations in influent and effluent TN concentrations and removal rate in the coupled system; (**b**) TN removal by different reactors.

As depicted in Figure 9a, the TN-removal efficiency of the coupled system showed an initial increase and then stabilized, reaching steady state after day 11 with an average removal efficiency of 82.22%. As illustrated in Figure 9b, after system stabilization, the average TN removal contribution rates of the SBABR and Anammox reactors were 24.31% and 57.91%, respectively. Results indicated that the majority of nitrogen removal was achieved in the Anammox reactor.

#### 3.2.2. Organic-Matter Removal Performance

**Figure 10:**
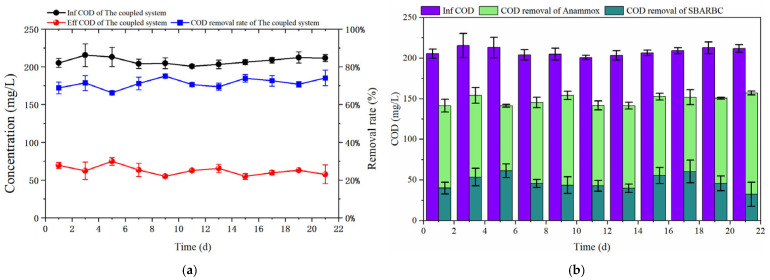
COD-removal analysis in the coupled system: (**a**) Variations in influent and effluent COD concentrations and removal rate; (**b**) COD removal by different reactors.

As shown in Figure 10a, the COD removal efficiency of the coupled system remained relatively stable with an average removal efficiency of 71.29%. As illustrated in Figure 10b, although the COD removal quantities of the SBABR and Anammox reactors exhibited fluctuation over time, the Anammox reactor consistently demonstrated higher COD removal than the SBABR reactor, with average removal contribution rates of 48.59% and 22.70%, respectively. This indicated that the anammox process played a dominant role in organic-matter removal.

COD removal in the SBABR reactor was attributed not only to aerobic degradation of organic matter by heterotrophic bacteria but also to direct organic matter uptake through algal assimilation [52]. Heterotrophic bacteria produced carbon dioxide during aerobic degradation of organic matter, which was assimilated by algae through photosynthesis to generate oxygen for heterotrophic bacteria, thereby enhancing organic matter removal. This formed a symbiotic relationship between heterotrophic bacteria and algae in the system [25]. COD removal in the Anammox reactor was primarily achieved through denitrification by denitrifying bacteria.

### 3.3. Effluent Color Characteristics

**Figure 11:**
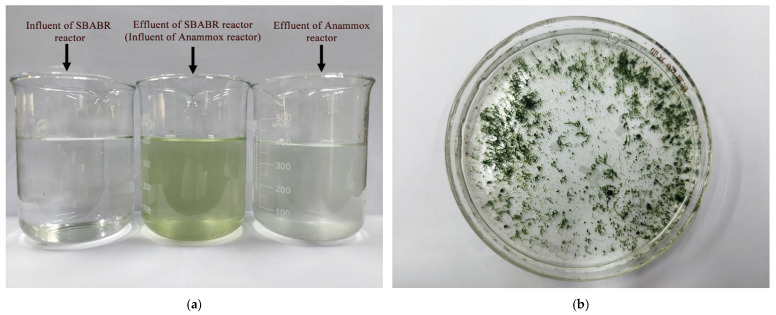
Effluent visual characteristics: (**a**) Color variation between influent and effluent; (**b**) Algal flocs in partial nitrification effluent.

As shown in Figure 11a, the SBABR reactor effluent exhibited a yellow-green color with noticeable turbidity. Detection results revealed an effluent chlorophyll-a concentration of 296.40 µg/L and color of 42 Hazen units. This was primarily attributed to the detachment of algae from the biofilm surface during disk rotation (as shown in Figure 11b), which resulted in effluent coloration [53].

When the SBABR reactor effluent was fed into the Anammox reactor for nitrogen removal, the effluent chlorophyll-a concentration decreased to below the limit of detection, with color reduced to only 17 Hazen units. As illustrated in Figure 11a, the Anammox reactor effluent appeared nearly colorless. The removal of chlorophyll-a and color through the anammox process has not been specifically reported in the previous literature [54,55,56,57,58]. This phenomenon may be attributed to the adsorption and interception of organic matter, including chlorophyll-a by biofilm in the Anammox reactor, as well as degradation by anaerobic microorganisms under anaerobic conditions [59].

Current methods for removing chlorophyll-a and color use pre-ozonation combined with coagulation [60], membrane filtration, and advanced oxidation processes [61]. This system achieved effective removal of chlorophyll-a and color through the anammox process while simultaneously removing nitrogen, without post-treatment processes such as coagulation, membrane filtration, or advanced oxidation. This treatment approach not only reduces costs but also provides a novel solution for removing chlorophyll-a from bacteria–algae symbiotic system effluents. However, during operational management, if the rotational speed is too low, excessive algal growth may occur. Excessive algal growth can shield microorganisms residing in the inner layers from sunlight, potentially creating niches for NOB growth in the deeper layers. This situation could disrupt partial nitrification, thereby affecting the nitrite nitrogen concentration in the Anammox influent.

### 3.4. Mechanisms of Light Inhibition on NOB and Algae Removal

#### 3.4.1. Mechanism of Light Inhibition on NOB for Achieving Partial Nitrification

Microbial-community analysis revealed that AOB had an abundance of 0.69% while NOB had only 0.01% abundance, yielding an abundance ratio of 69:1. As shown in Table 1, exposure to sunlight inhibited both AOB and NOB, with NOB experiencing a more pronounced inhibition. This differential inhibition resulted in an AOB/NOB abundance ratio significantly higher than values reported for conventional rotating biological contactor systems without sunlight (0.72–0.85 in Table 1), providing the biological foundation for stable partial nitrification. Correspondingly, the NAR increased from 34.00% [18] to above 95%. The underlying mechanisms are elucidated below from the perspective of light-induced bio-chemical effects.

Light exerts differential inhibitory effects on AOB and NOB, with NOB demonstrating significantly higher photosensitivity. Both groups contain photosensitive c-type cytochromes with absorption near 408 nm [62,63]. However, NOB are particularly vulnerable because light severely suppresses *nxrB* gene expression, reducing synthesis of nitrite oxidoreductase (NXR), the key enzyme for nitrite oxidation [64]. Blue light further inhibits NOB through blue light-utilizing flavin (BLUF) photoreceptors while AOB express enhanced DNA repair genes [65]. Additionally, photochemically produced reactive oxygen species contribute to photoinhibition [66].

The critical distinction lies in recovery capacity. AOB recover efficiently through ATP-dependent proteases that degrade photoinactivated AMO and enable synthesis of functional enzymes, with high NH_3_ concentrations providing additional photoprotection by promoting AMO synthesis [67]. In contrast, NOB exhibit markedly slower recovery [68]. This differential response—combining preferential gene suppression in NOB with re-pair and substrate-mediated protection in AOB—creates the selective pressure enabling stable partial nitrification under light exposure.

Beyond direct light effects, the system’s chemical environment synergistically promoted NOB inhibition. Algal photosynthesis consumed carbon dioxide, elevating system pH to 9.86 and free ammonia concentration to 80.87 mg/L. Since the inhibition threshold of free ammonia for NOB (0.1–1 mg/L) is significantly lower than for AOB (10–150 mg/L), high free ammonia concentrations further enhanced selective NOB inhibition [69]. The synergistic effects of light exposure and high free ammonia collectively maintained nitrite accumulation rates at 94.98–98.13%, achieving stable partial nitrification even under substantial light intensity fluctuations (300–2400 μmol/(m^2^·s)).

#### 3.4.2. Mechanism of Algae Removal in the Anammox Reactor

Section 3.3 showed that the Anammox reactor achieved significant decolorization effects, with substantial reductions in effluent color and chlorophyll-a concentration. This section systematically elucidates the mechanisms of algae removal from two aspects, physical retention and microbial degradation. The SBABR reactor effluent appeared yellow-green (color 42 Hazen units, chlorophyll-a concentration 296.40 µg/L), which was primarily attributed to detachment of algal cells from the biofilm surface. After treatment in the Anammox reactor, chlorophyll-a concentration decreased to below detection limits and color was reduced to 17 Hazen units, achieving nearly colorless effluent.

The Anammox reactor employed an upflow packed bed configuration. When SBABR effluent passed through the packing layer, algal cells were retained by the three-dimensional biofilm network. The biofilm surface was rich in extracellular polymeric substances (EPS) [70], which adsorbed and immobilized algal cells through electrostatic interactions, hydrogen bonding, and hydrophobic interactions [71].

The microbial composition provided abundant functional groups for algae degradation. Bacteroidetes (41.04% abundance) was the dominant phylum, possessing powerful extracellular hydrolytic enzyme systems capable of secreting cellulase and hemicellulose [72]. These enzymes disrupted the polysaccharide backbone of algal cell walls [73], causing cell lysis and pigment release [74]. Proteobacteria (10.84% abundance) and Firmicutes (6.96% abundance) further participated in organic compound degradation through fermentation and anaerobic respiration [75]. Studies have demonstrated that these three phyla effectively degrade algal chlorophyll under anaerobic conditions [76,77], consistent with their high abundance in this study and confirming that biological degradation by anaerobic microbial communities was the key decolorization mechanism.

## 4. Conclusions

This study successfully established a sunlight-enhanced bacteria–algae partial nitrification–anammox coupled system. Under conditions of significant light intensity fluctuation (300–2400 μmol/(m^2^·s)), the SBABR reactor maintained NAR above 95%. The research revealed that sunlight exerted stronger inhibition on NOB than on AOB, achieving a Nitrosomonas to Nitrospira abundance ratio of 69:1. Combined with the high pH (9.86) and the high FA (80.87 mg/L) environment generated by algal photosynthesis, stable partial nitrification was established. This method utilizing natural light avoids the complex control requirements of traditional processes, not only inhibiting NOB but also providing significant energy-saving advantages.

The coupled system demonstrated favorable pollutant removal performance, achieving average TN and COD removal efficiencies of 81.78% and 71.52%. Furthermore, this study discovered that the Anammox reactor exhibited removal capabilities for color and chlorophyll-a from the bacteria–algae system effluent (color reduced from 42 to 17 Hazen units, chlorophyll-a decreased below detection limits). This provides a novel approach for addressing color and chlorophyll-a issues in bacteria–algae symbiotic system effluents, offering reference for the engineering application of this technology.

## Data Availability

The raw data supporting the conclusions of this article will be made available by the authors on request.

## Abbreviations

The following abbreviations are used in this manuscript:

AN	Anammox reactor samples
Anammox	Anaerobic Ammonium Oxidation
AnAOB	Anaerobic Ammonium-Oxidizing Bacteria
AOB	Ammonia-Oxidizing Bacteria
APHA	American Public Health Association
ARE	Ammonia nitrogen Removal Efficiency
COD	Chemical Oxygen Demand
DNA	Deoxyribonucleic Acid
DO	Dissolved Oxygen
FA	Free Ammonia
FNA	Free Nitrous Acid
LED	Light-Emitting Diode
NAR	Nitrite Accumulation Rate
NOB	Nitrite-Oxidizing Bacteria
pH	Potential of Hydrogen
PN	Partial Nitrification reactor samples
PN/A	Partial Nitrification/Anaerobic ammonium oxidation
rRNA	Ribosomal Ribonucleic Acid
SBABR	Sunlight-enhanced Bacteria-Algae Biological Rotor
TN	Total Nitrogen
UV	Ultraviolet

## References

[1] Strokal M., Bai Z., Franssen W., Hofstra N., Koelmans A.A., Ludwig F., Ma L., van Puijenbroek P., Spanier J.E., Vermeulen L.C. (2021). Urbanization: An increasing source of multiple pollutants to rivers in the 21st century. Npj Urban Sustain..

[2] Zhang K., Liu X.-Y., Song W., Hien T.T., Wang X., Chen Z., Hai H.T.N., He S. (2023). Precipitation records of anthropogenic nitrogen pollution in two metropolitan cities of Southeast Asia. Urban Clim..

[3] Jin D., Zhang X., Zhou L., Zhang X., Wu P. (2024). Emerging applications and mechanisms of algal-bacterial symbiosis on sustainable wastewater treatment: A comprehensive review. J. Water Process Eng..

[4] Arp D.J., Stein L.Y. (2008). Metabolism of Inorganic N Compounds by Ammonia-Oxidizing Bacteria. Crit. Rev. Biochem. Mol. Biol..

[5] Strous M., Heijnen J.J., Kuenen J.G., Jetten M.S.M. (1998). The sequencing batch reactor as a powerful tool for the study of slowly growing anaerobic ammonium-oxidizing microorganisms. Appl. Microbiol. Biotechnol..

[6] Zhao Q., Peng Y., Li J., Jia T., Zhang Q., Zhang L. (2024). Pilot-scale implementation of mainstream anammox for municipal wastewater treatment against cold temperature. Nat. Commun..

[7] Chen X., Liu L., Bi Y., Meng F., Wang D., Qiu C., Wang C., Wang S., Zhang B. (2024). Preservation and reactivation of anammox biomass: A mini review. J. Environ. Chem. Eng..

[8] Zhou W., Zhang Q., Wang B., Hou F., Pang H., Guo Y., Zhang L., Peng Y. (2025). Temperature-based strategy for enhanced nitrogen removal in mainstream via selectively strengthening anammox or denitrification. Npj Clean Water.

[9] Gu S., Wang S., Yang Q., Yang P., Peng Y. (2012). Start up partial nitrification at low temperature with a real-time control strategy based on blower frequency and pH. Bioresour. Technol..

[10] Liu X., Kim M., Nakhla G., Andalib M., Fang Y. (2020). Partial nitrification-reactor configurations, and operational conditions: Performance analysis. J. Environ. Chem. Eng..

[11] Guo G., Zhou S., Chen Y., Li Y.-Y. (2024). Phosphorus recovery coupling with one-stage partial nitritation/anammox process for the treatment of high-nutrient permeate from anaerobic membrane bioreactor treating concentrated organic sludge. Chem. Eng. J..

[12] Wang W., Jiang T., Wang S., Wang L., Li Z., Li W., Wang B. (2024). Low alkalinity, free ammonia, and free nitrous acid cooperatively stabilize partial nitrification under excessive aeration condition. Chemosphere.

[13] Miao Y., Zhang L., Yu D., Zhang J., Zhang W., Ma G., Zhao X., Peng Y. (2022). Application of intermittent aeration in nitrogen removal process: Development, advantages and mechanisms. Chem. Eng. J..

[14] Duan C., Zhang Q., Li J., Feng W., Zhang L., Peng Y. (2024). Partial nitrification response to dissolved oxygen variation and aerobic starvation: Kinetics and microbial community analyses. Chem. Eng. J..

[15] Wang L., Kang X., Liu Y., Huang X. (2023). Free ammonia-free nitrous acid based partial nitrification in sequencing batch membrane aerated biofilm reactor. Water Res..

[16] Lu S., Li Y., Liu X., Cheng G., Yuan Z., Wu F. (2023). Influence of Light Irradiation on Nitrification in Microalgal–Bacterial Systems for Treating Wastewater. Processes.

[17] Si G., Liu B., Liu Y., Yan T., Wei D. (2022). Light-introduced partial nitrification in an algal-bacterial granular sludge bioreactor: Performance evolution and microbial community shift. Bioresour. Technol..

[18] Yan Z., Pei Z. (2024). Light Enables Partial Nitrification and Algal-Bacterial Consortium in Rotating Biological Contactors: Performance and Microbial Community. Sustainability.

[19] Chu Z., Huang D., Huang X., He J., Chen L., Wang J., Rong H. (2023). Achieving robust mainstream nitritation by implementing light irradiation: Long-term performance and microbial dynamics. Bioresour. Technol..

[20] Huang W., Liu D., Huang W., Cai W., Zhang Z., Lei Z. (2020). Achieving partial nitrification and high lipid production in an algal-bacterial granule system when treating low COD/NH4–N wastewater. Chemosphere.

[21] Kim K., Park Y.-G. (2021). Light as a Novel Inhibitor of Nitrite-Oxidizing Bacteria (NOB) for the Mainstream Partial Nitrification of Wastewater Treatment. Processes.

[22] Meng Q., Zeng W., Liu H., Zhan M., Zhang J., Wu H. (2023). The successful application of light to the system of simultaneous nitrification/endogenous denitrification and phosphorus removal: Promotion of partial nitrification and glycogen accumulation metabolism. Water Res..

[23] Zheng X., Liu R., Li K., Sun J., Wang K., Shao Y., Hu Z., Zhu J., Pan Z., Nakhla G. (2025). Microalgae-bacteria symbiosis enhanced nitrogen removal from wastewater in an inversed fluidized bed bioreactor: Performance and microflora. Front. Microbiol..

[24] Xin C., Khu S.-T., Wang T., Zuo X., Zhang Y. (2024). Effect of flow fluctuation on water pollution in drinking water distribution systems. Environ. Res..

[25] Chen J., Liu X., Lu T., Liu W., Zheng Z., Chen W., Yang C., Qin Y. (2024). The coupling of anammox with microalgae-bacteria symbiosis: Nitrogen removal performance and microbial community. Water Res..

[26] Huang L., Lu Z., Xie T., Wang L., Mo C. (2022). Nitrogen and phosphorus removal by coupling Anaerobic ammonia oxidation reaction with algal-bacterial symbiotic system. Water Res..

[27] Yang M., Xie K.-P., Ma C., Yu S.-H., Ma J.-Y., Yu Z.-Q., Chen X., Gong Z. (2022). Achieving partial nitrification-anammox process dependent on microalgal-bacterial consortia in a photosequencing batch reactor. Front. Bioeng. Biotechnol..

[28] Zhang Y., Wang J., Peng S., Zhao D., Miao L. (2022). Autotrophic biological nitrogen removal in a bacterial-algal symbiosis system: Formation of integrated algae/partial-nitrification/anammox biofilm and metagenomic analysis. Chem. Eng. J..

[29] APHA, AWWA, WEF (2017). Part 4000 Inorganic Nonmetallic Constituents. Standard Methods For the Examination of Water and Wastewater.

[30] Jiao N., Liu J., Edwards B., Lv Z., Cai R., Liu Y., Xiao X., Wang J., Jiao F., Wang R. (2021). Correcting a major error in assessing organic carbon pollution in natural waters. Sci. Adv..

[31] (2017). 2120 COLOR. Standard Methods For the Examination of Water and Wastewater.

[32] Jeffrey S.W., Humphrey G.F. (1975). New spectrophotometric equations for determining chlorophylls a, b, c1 and c2 in higher plants, algae and natural phytoplankton. Biochem. Physiol. Pflanz..

[33] Liu Y., Zhu Y., Wu D., Wang Z., Wang Y., Wang G., Zhou X., Sun H. (2023). Effect of free nitrous acid on nitritation process: Microbial community, inhibitory kinetics, and functional biomarker. Bioresour. Technol..

[34] Zheng Y., Shi L., Su R., Min W., Wei Y., Ma B. (2025). Effects of periodic starvation on the characteristics and microbial communities of anammox sludge. J. Environ. Chem. Eng..

[35] Anthonisen A.C., Loehr R.C., Prakasam T.B.S., Srinath E.G. (1976). Inhibition of Nitrification by Ammonia and Nitrous Acid. J. (Water Pollut. Control Fed.).

[36] Oon Y.-S., Oon Y.-L., Ayaz M., Wang Y., Deng M., Li L., Song K., Wu F. (2025). Microalgae-bacteria synergy in photosynthetic bio-electrochemical systems supports nitrogen transformation, microbial dynamics and greenhouse gas mitigation. Commun. Earth Environ..

[37] Verbaendert I., Boon N., De Vos P., Heylen K. (2011). Denitrification is a common feature among members of the genus Bacillus. Syst. Appl. Microbiol..

[38] Yang X., Hu F., Qin J., Pang X., Zhang Y., Ji J. (2025). The establishment of a continuous-flow partial nitrification biofilm system via hydroxylamine dosing. Chem. Eng. J..

[39] Matsakas L., Furnish B.J., Keller T.A. (2020). Carbon limitation in hypereutrophic, periphytic algal wastewater treatment systems. Plos One.

[40] Chain P., Lamerdin J., Larimer F., Regala W., Lao V., Land M., Hauser L., Hooper A., Klotz M., Norton J. (2003). Complete Genome Sequence of the Ammonia-Oxidizing Bacterium and Obligate Chemolithoautotroph *Nitrosomonas europaea*. J. Bacteriol..

[41] Daims H., Lücker S., Wagner M. (2016). A New Perspective on Microbes Formerly Known as Nitrite-Oxidizing Bacteria. Trends Microbiol..

[42] Vergara C., Muñoz R., Campos J.L., Seeger M., Jeison D. (2016). Influence of light intensity on bacterial nitrifying activity in algal-bacterial photobioreactors and its implications for microalgae-based wastewater treatment. Int. Biodeterior. Biodegrad..

[43] Dong Y., Guo J., Zhong Z., Wang J., Chen Y. (2021). Packed cage rotating biological contactor for mustard tuber wastewater treatment: Performance and microbiome along the axial direction. J. Water Process Eng..

[44] Wang D., Wang Y., Liu L., Chen Y., Wang C., Xu X., Yang Y., Wang Y., Zhang T. (2022). Niche differentiation and symbiotic association among ammonia/nitrite oxidizers in a full-scale rotating biological contactor. Water Res..

[45] Hu N., He J., Shi W., He J., Lv B., Liang Y., Huang L. (2022). Ecological restoration for the Liangtan river by Rotating biological contactors combined with hybrid constructed wetlands. J. Clean. Prod..

[46] (1996). Ministry of Ecology and Environment.

[47] Strous M., Pelletier E., Mangenot S., Rattei T., Lehner A., Taylor M.W., Horn M., Daims H., Bartol-Mavel D., Wincker P. (2006). Deciphering the evolution and metabolism of an anammox bacterium from a community genome. Nature.

[48] Mardanov A.V., Beletsky A.V., Ravin N.V., Botchkova E.A., Litti Y.V., Nozhevnikova A.N. (2019). Genome of a Novel Bacterium “Candidatus Jettenia ecosi” Reconstructed From the Metagenome of an Anammox Bioreactor. Front. Microbiol..

[49] Eng Nkonogumo P.L., Zhu Z., Emmanuel N., Zhang X., Zhou L., Wu P. (2024). Novel and innovative approaches to partial denitrification coupled with anammox: A critical review. Chemosphere.

[50] Marchant H.K., Ahmerkamp S., Lavik G., Tegetmeyer H.E., Graf J., Klatt J.M., Holtappels M., Walpersdorf E., Kuypers M.M.M. (2017). Denitrifying community in coastal sediments performs aerobic and anaerobic respiration simultaneously. ISME J..

[51] Fahrbach M., Kuever J., Meinke R., Kämpfer P., Hollender J. (2006). Denitratisoma oestradiolicum gen. nov., sp. nov., a 17β-oestradiol-degrading, denitrifying betaproteobacterium. Int. J. Syst. Evol. Microbiol..

[52] Perez-Garcia O., Escalante F.M.E., de-Bashan L.E., Bashan Y. (2011). Heterotrophic cultures of microalgae: Metabolism and potential products. Water Res..

[53] Xing Z., Siqi L., Shouguo Y., Xingzhi Z., Ely V.H., Zhifeng G., Aimin W. (2021). Change and correlation analysis of pigment contents and color value during growth of Chlorella vulgaris. South China Fish. Sci..

[54] Luo Q., Li Y.-s., Chang B.-z., Zhang S., Chen D.-z., Jin R.-c., Yang G.-f. (2025). Response of biofilm and granular sludge to low dissolved oxygen and organics in a single anammox reactor: Performance, bacterial community and function genes. Sci. Total Environ..

[55] Ni L., Wang P., Zhou G., Li Z., Wang Y. (2025). In situ electrocatalytic nitrate-to-nitrite conversion-driven anammox in MBRs for extremely efficient ammonium-containing wastewater treatment. Water Res..

[56] Wang S., Dai B., Wang Z., Yang S., Xia S. (2025). Sulfur-driven partial denitrification coupled with anammox: Advances, optimization, and engineering prospects for sustainable nitrogen removal. J. Environ. Chem. Eng..

[57] Chi Y., Ren W., Jin P., Shi X., Liu L. (2025). Robust partial nitrification and anammox under low-strength nitrogen condition by regulating organic-induced symbiosis of denitrifiers and anammox bacteria. Bioresour. Technol..

[58] Guo Y., Lei X., Wang Z., Fu Z., Zhou Y., Liu Q., Li K., Fu M. (2025). The influence of Fe (II) on anammox process under low temperature: Microbial community and predictive functional profiling. J. Environ. Chem. Eng..

[59] Zhang L., Peng Y., Soda S., Huang X., Wang Y., Zhang Y. (2020). Molecular-level characterization of stratified extracellular polymeric substances of anammox sludge and its adsorption preference to refractory dissolved organic matter. Energy.

[60] Masoomi B., Jaafarzadeh N., Tabatabaie T., Kouhgardi E., Jorfi S. (2019). Effects of pre-ozonation and chemical coagulation on the removal of turbidity, color, TOC, and chlorophyll a from drinking water. Environ. Health Eng. Manag..

[61] Collivignarelli M.C., Abbà A., Carnevale Miino M., Damiani S. (2019). Treatments for color removal from wastewater: State of the art. J. Environ. Manag..

[62] Wang L., Qiu S., Guo J., Ge S. (2021). Light Irradiation Enables Rapid Start-Up of Nitritation through Suppressing nxrB Gene Expression and Stimulating Ammonia-Oxidizing Bacteria. Environ. Sci. Technol..

[63] Guerrero M.A., Jones R.D. (1997). Light-induced absorbance changes associated with photoinhibition and pigments in nitrifying bacteria. Aquat. Microb. Ecol..

[64] Neissi A., Rafiee G., Rahimi S., Farahmand H., Pandit S., Mijakovic I. (2022). Enriched microbial communities for ammonium and nitrite removal from recirculating aquaculture systems. Chemosphere.

[65] Zheng R., Feng Y., Kong L., Wu X., Zhou J., Zhang L., Liu S. (2024). Blue-light irradiation induced partial nitrification. Water Res..

[66] Gottshall E.Y., Godfrey B., Li B., Abrahamson B., Qin W., Winkler M. (2022). Photoinhibition of comammox reaction in Nitrospira inopinata in a dose- and wavelength-dependent manner. Front. Microbiol..

[67] Lu S., Liu X., Liu C., Cheng G., Shen H. (2020). Influence of photoinhibition on nitrification by ammonia-oxidizing microorganisms in aquatic ecosystems. Rev. Environ. Sci. Bio/Technol.

[68] Merbt S.N., Stahl D.A., Casamayor E.O., Martí E., Nicol G.W., Prosser J.I. (2012). Differential photoinhibition of bacterial and archaeal ammonia oxidation. FEMS Microbiol. Lett..

[69] Li Y., Chen Z., Zhang Y., Wang Z., Zhang C., Deng Z., Huang L., Wang X., Fan J., Zhou S. (2023). Response of partial nitritation and denitrification processes to high levels of free ammonia in a pilot mature landfill leachate treatment system: Stability and microbial community dynamics. Bioresour. Technol..

[70] Costa O.Y.A., Raaijmakers J.M., Kuramae E.E. (2018). Microbial Extracellular Polymeric Substances: Ecological Function and Impact on Soil Aggregation. Front. Microbiol..

[71] Tong C.Y., Lew J.K., Derek C.J.C. (2022). Algal extracellular organic matter pre-treatment enhances microalgal biofilm adhesion onto microporous substrate. Chemosphere.

[72] El Houari A., Carpenter M., Chaplin D., Golyshin P., McDonald J.E. (2025). Taxonomic description and genome sequence of Anaerorudis cellulosivorans gen. Nov. sp. nov., a novel cellulose- and Xylan-degrading bacterium of the Bacteroidota phylum isolated from a lab-scale methanogenic landfill bioreactor digesting municipal solid waste. Syst. Appl. Microbiol..

[73] Passos F., Hom-Diaz A., Blanquez P., Vicent T., Ferrer I. (2016). Improving biogas production from microalgae by enzymatic pretreatment. Bioresour. Technol..

[74] Safi C., Frances C., Ursu A.V., Laroche C., Pouzet C., Vaca-Garcia C., Pontalier P.-Y. (2015). Understanding the effect of cell disruption methods on the diffusion of Chlorella vulgaris proteins and pigments in the aqueous phase. Algal Res..

[75] Llamas M., Magdalena J.A., Greses S., Tomás-Pejó E., González-Fernández C. (2021). Insights on the microbial communities developed during the anaerobic fermentation of raw and pretreated microalgae biomass. Chemosphere.

[76] Morrison J.M., Murphy C.L., Baker K., Zamor R.M., Nikolai S.J., Wilder S., Elshahed M.S., Youssef N.H. (2017). Microbial communities mediating algal detritus turnover under anaerobic conditions. PeerJ.

[77] Xing P., Guo L., Tian W., Wu Q.L. (2011). Novel Clostridium populations involved in the anaerobic degradation of Microcystis blooms. ISME J..

